# The T-cell receptor β chain CDR3 insights of bovine liver immune repertoire under heat stress

**DOI:** 10.5713/ab.24.0152

**Published:** 2024-06-25

**Authors:** Linhu Hui, Fengli Wu, Yuanyuan Xu, Guangjun Yang, Qiaorong Luo, Yangyang Li, Long Ma, Xinsheng Yao, Jun Li

**Affiliations:** 1 Department of Immunology, Center of Immunomolecular Engineering, Innovation & Practice Base for Graduate Students Education, Zunyi Medical University, Zunyi 563000, China; 2 Xiangyun County Livestock Workstation, Xiangyun 671000, China; 3 College of Veterinary Medicine, Yunnan Agricultural University, Kunming 650201, China

**Keywords:** Bovine Liver, Heat Stress, Immune Repertoire, T-cell Receptor (TCR) β Chain, Tissue-specific T Cells

## Abstract

**Objective:**

The liver plays a dual role in regulating temperature and immune responses. Examining the influence of heat stress (HS) on liver T cells contributes significantly to understanding the intricate interplay between the immune system and hepatic tissues under thermal stress. This study focused on investigating the characteristics of the T-cell receptor (TCR) β chain CDR3 repertoire in bovine liver samples under both HS and pair-fed (PF) environmental conditions.

**Methods:**

Sequencing data from six samples sourced from the GEO database underwent annotation. Utilizing immunarch and VDJtool software, the study conducted comprehensive analyses encompassing basic evaluation, clonality assessment, immune repertoire comparison, diversity estimation, gene usage profiling, VJ gene segment pairing scrutiny, clonal tracking, and Kmers analysis.

**Results:**

All four TCR chains, namely α, β, γ, and δ, were detected, with the α chains exhibiting the highest detection frequency, followed closely by the β chains. The prevalence of αβ TCRs in bovine liver samples underscored their crucial role in governing hepatic tissue’s physiological functions. The TCR β CDR3 repertoire showcased substantial inter-individual variability, featuring diverse clonotypes exhibiting distinct amino acid lengths. Intriguingly, HS cattle displayed heightened diversity and clonality, suggesting potential peripheral T cell migration into the liver under environmental conditions. Notably, differential VJ gene pairings were observed in HS cattle compared to the PF, despite individual variations in V and J gene utilization. Additionally, while most high-frequency amino acid 5-mers remained consistent between the HS and PF, GELHF, and YDYHF were notably prevalent in the HS group. Across all samples, a prevalent trend of high-frequency 5mers skewed towards polar and hydrophobic amino acids was evident.

**Conclusion:**

This study elucidates the characteristics of liver TCR β chain CDR3 repertoire under HS conditions, enhancing our understanding of HS implications.

## INTRODUCTION

Heat stress (HS) poses a significant challenge in dairy farming, potentially exerting adverse effects on the health, reproductive capabilities, and immune system of dairy cows [1,2]. This stress condition markedly reduces both the overall milk yield and its quality, concurrently triggering inflammatory responses and immune system dysregulation within the cows, severely impacting their immune response and overall physiological status. The liver, as a pivotal metabolic hub, plays a crucial role in regulating energy balance and coping with additional heat load [3]. Heat stress prompts the organism to manage surplus heat, elevating the metabolic rate and subsequently affecting liver function [4]. The liver, under these circumstances, assumes responsibility for regulating energy metabolism and distributing heat, adjusting pathways such as glucose and lipid metabolism along with heat production to adapt to elevated temperatures. Moreover, the liver as an immunogenic organ is exposed to a variety of pathogens entering the liver through the systemic blood circulation or via the portal vein from the gut [5]. Hence, comprehensively understanding the interplay between the immune system and the liver under conditions of HS is paramount in elucidating the impact of HS on immune responses.

Tissue-specific T cells play a crucial role in maintaining local immune homeostasis and responding to tissue infections, providing protective immunity. Tissue-specific T cells undertake surveillance within the tissue to encounter their specific antigen, thus establishing effective protection against secondary infections [6]. Unlike T cells in the circulatory system, tissue-specific T cells exhibit unique functionalities and regulatory characteristics due to their distinct positioning within specific tissues [7]. For instance, skin-specific T cells promptly respond to external stimuli, playing a pivotal role in immune protection [8]. Intestinal-specific T cells contribute to regulating intestinal mucosal immune balance and preserving gut health [9]. Liver-specific T cells hold a distinctive role in various liver conditions, including hepatitis B and C infections [10], non-alcoholic fatty liver disease [11], and even contribute to establishing persistent immune responses post-hepatitis vaccination or in the progression of hepatocellular carcinoma [12]. However, the impact of HS on the homeostasis of T cells within liver tissue remains unclear.

The specificity of T cells relies on the composition of their surface receptor (TCR), which recognize and bind to antigen peptide-MHC complexes, initiating crucial signaling events essential for T cell immune response [13]. The T-cell receptor (TCR) consists of α and β (or γ and δ) chains, and the collective information of all individual TCR sequences constitutes the TCR repertoire. According to the allelic exclusion theory, each T cell surface expresses only one type of TCR, forming the molecular basis for T cell specificity [14]. Thus, in-depth exploration of TCR repertoire diversity, variability, and their roles in antigen recognition and immune response enables a comprehensive understanding of how T cells engage in immune responses [15]. In this study, we investigated the TCR repertoire present in the liver tissue of heat-stressed cattle. Our aim was to uncover the diversity and variability of tissue-resident T cells under HS conditions. This exploration significantly contributes to our understanding of how HS affects the immune response and immunological adaptability within the cattle liver.

## MATERIALS AND METHODS

### Data acquisition and preprocessing

This study incorporated a total of six bovine liver tissue samples for immune repertoire analysis, comprising three samples from heat-stressed environmental conditions (6 am to 6 pm 27°C to 37°C, THI = 74 to 82; 6 pm to 6 am 27°C, THI = 74; n = 3, average days in milk = 211) and three from the pair-fed environmental conditions (match the feed intake of heat stressed cows, n = 3, average days in milk = 226). The definition of HS in our study is based on the work by Li et al [16]. Transcriptome sequencing data for these six samples were obtained from the GEO database under the accession number GSE226351, stored in FASTQ format. To ensure data quality, a rigorous quality control process was employed using FASTQC tools. This process encompassed sequence quality assessment, removal of adapter sequences and low-quality reads, followed by filtering and trimming of reads to prepare them for subsequent analysis.

### Annotation of immune cell receptors

The IMGT-provided bovine immune cell receptor reference database was chosen as the background reference for our analysis. Using the MiXCR software (https://mixcr.com/) [17], we performed annotation on the preprocessed sequencing data according to the instruction. This step aimed to identify and analyze relevant information regarding TCR β chains, including genes and sequences, facilitating subsequent analysis of the TCR CDR3 repertoire.

### Analysis of T-cell receptor β chains

For a comprehensive analysis of TCR β chain characteristics, we utilized the immunarch package in R and the VDJtool software [18]. Initially, basic analysis and clonality assessments were conducted to evaluate statistical features and clonality of TCR β chains in each sample. Subsequently, comparisons were made regarding the repertoire overlap between different samples, investigating shared clonotypes. We further assessed the diversity of TCR β chains and compared diversity discrepancies between the heat-stressed and control groups. Additionally, an analysis of gene usage and pairing frequencies of VJ gene segments was performed to understand their distribution within the TCR β chains. Finally, clonotype tracking and Kmers analysis were conducted to explore sequence features and variations.

### Data statistics and visualization

Statistical analysis was performed using GraphPad Prism v5.0. Statistical significance was evaluated using a one-way analysis of variance with a Bonferroni post-test *** p<0.001, ** p<0.01, * p<0.05, ns = not significance (p>0.05). To present the results and findings clearly, visual representations in the form of plots and graphs were generated using the ggplot2 package in R.

## RESULTS

### Quantitative analysis of immune cell receptor chains

T cells are categorized into αβ TCR T cells and γδ TCR T cells based on their distinct receptors, comprising α and β chains or γ and δ chains, respectively [19]. All four types of TCR chains were detected in bovine liver samples (Table 1), with α chains showing the highest detection frequency, followed by β chains. While γ and δ chains were present in all samples, their quantities were notably lower than α and β chains. These findings suggest that although γδTCR T cells play a vital role in ruminants, hepatic αβ tissue-resident T cells may be more prominently involved in regulating the physiological functions of bovine liver tissue. This presence of αβ T cells potentially contributes to the modulation of immune responses and functionalities within bovine hepatic tissues.

Furthermore, we detected the heavy chain and two types of light chains of the immunoglobulin receptor, all of which were present in greater quantities compared to the four chains of the TCR (Table 1). Following the established rules of B cell receptor rearrangement, the heavy chain undergoes rearrangement initially, followed by rearrangement of the κ chain within the light chains [20]. It’s noteworthy that in humans and mice, the rearrangement of light chains does not adhere to the allelic exclusion rule, resulting in a higher quantity of κ chains than heavy chains, with the lowest count observed for λ chains [21]. However, our study observed that in bovine liver tissues, the quantity of λ chains was the highest, while κ chains were the least abundant. This suggests a potential divergence in the rearrangement pattern of B cell receptors in cattle compared to humans and mice.

### Basic analysis and clonality of bovine T-cell receptor β CDR3 repertoire

**Figure 1:**
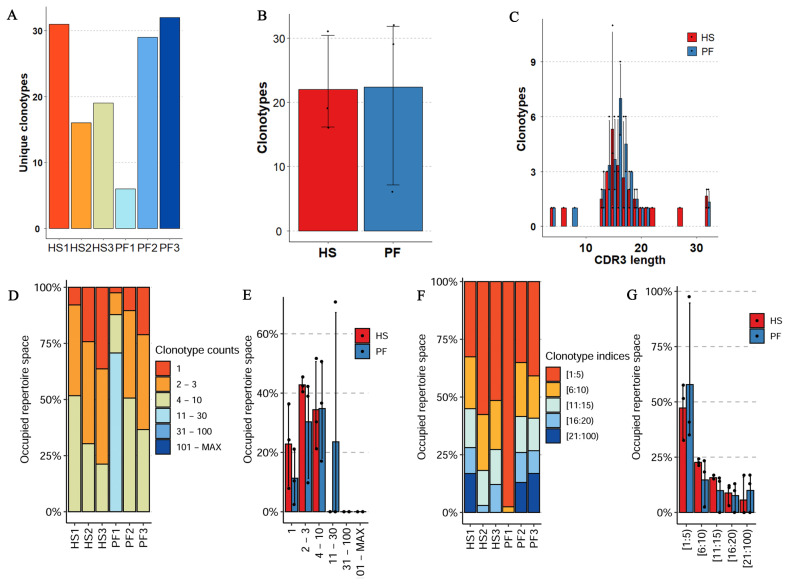
Representation of TCR β chain clones detected in bovine liver tissue. (A) Number of unique clonotypes for 6 samples; (B) differential analysis of clonotypes between HS and PF cattle; (C) the length distribution of CDR3; (D) the rare clonal proportion; (E) the difference of rare clonal proportion with specific count between HS and PF cattle; (F) the top clonal proportion; (G) the difference of top clonal proportion with specific indices between HS and PF cattle. TCR, T-cell receptor; HS, heat stressed cattle; PF, pair-fed cattle. The number of samples for HS and PF groups in B, C, and G is n = 3.

The basic analysis and clonality assessment of the bovine TCR β CDR3 repertoire aimed to characterize the diversity and distribution of clonotypes under heat HS and PF conditions. A total of 133 unique clonotypes were detected, comprising 66 in the HS group and 67 in the PF group (Figure 1A). Considerable inter-individual variability was observed, although no statistically significant inter-group differences were identified (Figure 1B). Analysis of the clonotypes’ CDR3 lengths revealed a predominant distribution within the range of 13 to 21 amino acids. Peaks were observed at 15 amino acids for the HS group and at 16 amino acids for the PF group; however, the abundance of clonotypes at both 15 and 16 amino acids did not significantly differ between the HS and PF groups (Figure 1C). Subsequently, clonality in the two sample sets was further analyzed, encompassing Top and rare clonal proportions (Figure 1D–1G). The HS group exhibited a greater presence of rare clonotypes compared to the PF group, while high-frequency clonotypes predominantly occupied the repertoire space in the PF group (>50%).

### Repertoire overlap and public clonotypes of bovine T-cell receptor β CDR3 repertoire

**Figure 2:**
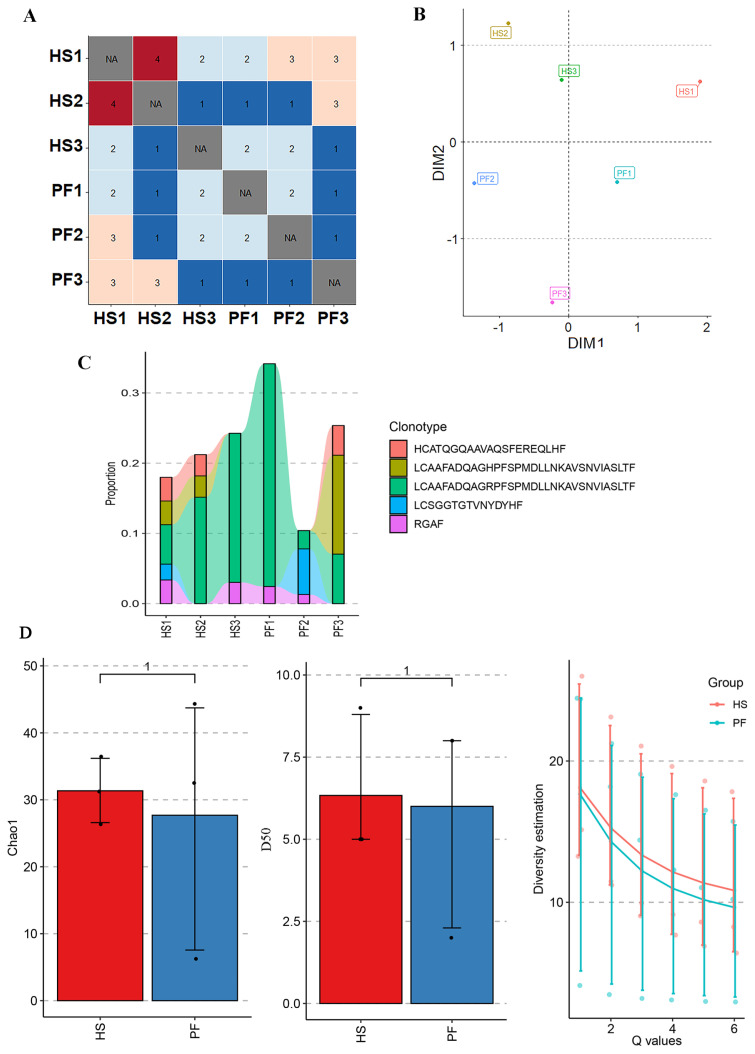
The similarity, public clonotypes and diversity of cattle liver TCR β CDR3 repertoire. (A) The repertoire similarity measured by repertoire overlap; (B) dimensionality reduction analysis based on rep-ertoire similarity; (C) tracking analysis of shared clones; (D) the estimation of repertoire diversity im-plemented in Chao, D50 and Hill index. TCR, T-cell receptor; HS, heat stressed cattle; PF, pair-fed cattle. The number of samples for HS and PF groups in D is n = 3.

Repertoire overlap and public clonotypes are crucial in understanding immune responses, as they reveal shared T-cell receptor sequences among individuals, providing insights into common adaptive immune responses. In the analysis of repertoire overlap and shared clones across samples, a total of five shared clones were detected across two or more samples, with a maximum coverage of five samples. The shared clones were relatively concentrated within the HS group (Figure 2A). Analysis based on the Diversity Index of the Morisita Index (Figure 2B) revealed distinct differences between the two groups along the DIM1 dimension, potentially associated with exposure to HS in cattle environments. However, there appeared to be similarity or overlap along the DIM2 dimension, possibly attributed to common features among liver-resident T-cell populations.

A tracking of the five shared clones across six samples (Figure 2C) highlighted variations in the clone “LCAAFAD QAGRPFSPMDLLNKAVSNVIASLTF” among the detected samples, consistently existing as multiclonal forms across these samples, with an average clone count of 4.8. In further estimation of repertoire diversity, incorporating Chao, D50, and Hill indices analysis (Figure 2D) for TCR β CDR3 repertoire in HS and PF cattle, although no significant differences were observed, all three indices indicated a higher diversity within the HS group compared to the PF group.

### Gene utilization and diversity assessment of bovine T-cell receptor β CDR3 repertoire

**Figure 3:**
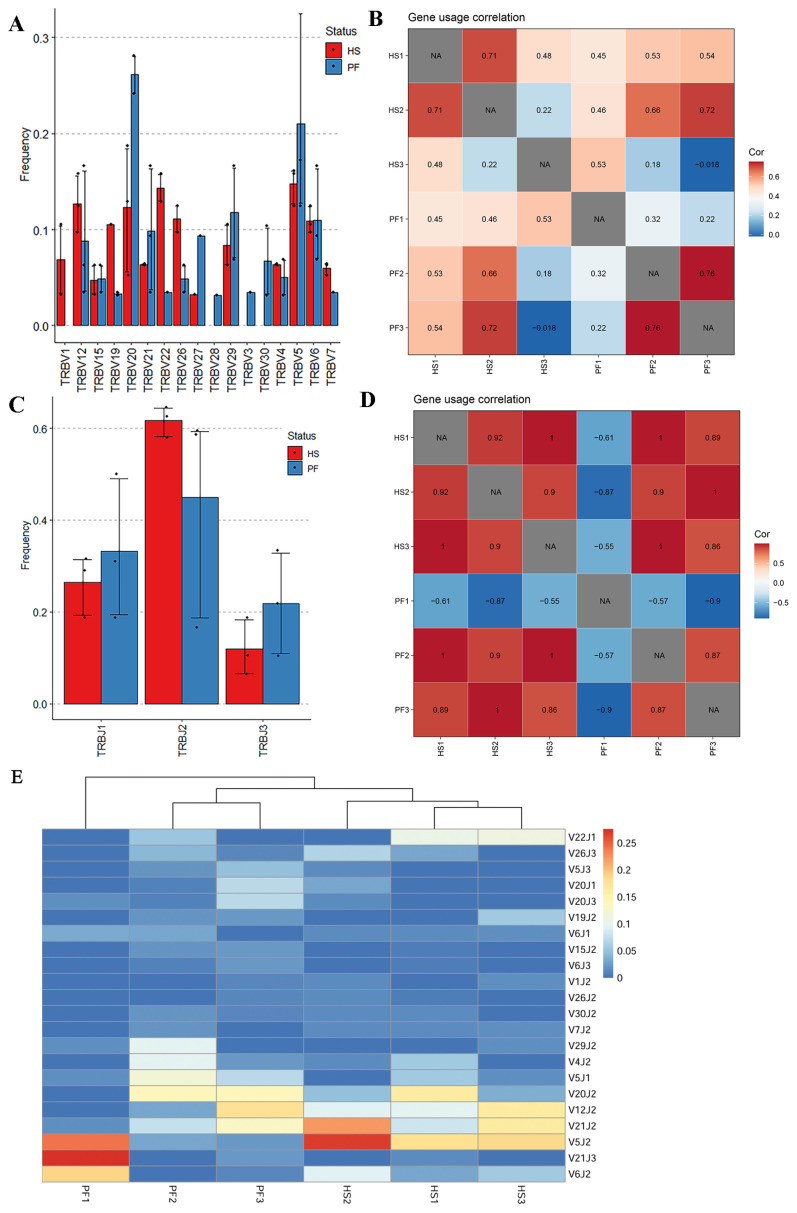
The usage and paring of V and J genes in cattle liver TCR β CDR3 repertoire. (A) statistical analysis of the V gene family usage in the HS and PF groups; (B) the correlation of V gene usage among all samples; (C) statistical analysis of the J gene family usage in the HS and PF groups; (D) the correlation of J gene usage among all samples; (E) heatmap of V-J gene pairings detected in at least three samples. TCR, T-cell receptor; HS, heat stressed cattle; PF, pair-fed cattle. The number of samples for HS and PF groups in A and C is n = 3.

The utilization and combination of V/J genes determine the clonal diversity and structural characteristics of TCR β chains, influenced by individual genetic background and environmental factors. Analyzing the utilization patterns of VJ genes enables the identification of potential immunological biomarkers or clonal groups associated with HS status. According to IMGT records, the bovine TCR β germline genes encompass 121 V genes and 19 J genes, categorized into 28 V gene families and 3 J gene families based on sequence homology. In this study, 17 out of the 28 V gene families were detected in bovine liver T cells (Figure 3A). While several V genes were only observed in specific groups of cattle, given the limited sample size, direct associations between these V genes and HS in cattle cannot be conclusively determined. Among the remaining utilized V genes, no significant differences were found between the two groups. Both groups exhibited relatively high-frequency utilization of the V20 and V5 gene families. The increased frequency of the V12 gene family potentially linked to its involvement with the shared clone “LCAAFADQAGRPFSPMDLLNKAVSNVIASLTF,” which averaged a clone count of 4.8 across five samples. Additionally, correlation analyses between V gene utilization and samples indicated inter-group differences, but primarily reflecting commonalities among individuals (Figure 3B).

All three J gene families were detected, with J2 being the most utilized J gene, also notably the only J gene family with a utilization rate higher in the HS group compared to the PF group (Figure 3C). Correlation analyses revealed strong associations (correlation coefficients >0.85) between the utilization of J gene families and all samples except PF1 (Figure 3D).

TCR β CDR3 sequences, formed through V(D)J gene rearrangements, encode the functional peptide chains, emphasizing the importance of analyzing VJ gene pairings in elucidating the ultimate TCR repertoire characteristics. Filtering for VJ pair information present in at least three samples, analysis revealed inter-group differences in VJ gene pairings, notably observed in V5J2, V21J2, and V22J1 pairings (Figure 3E).

### Kmers and sequence motif analysis of bovine T-cell receptor β CDR3 repertoire

**Figure 4:**
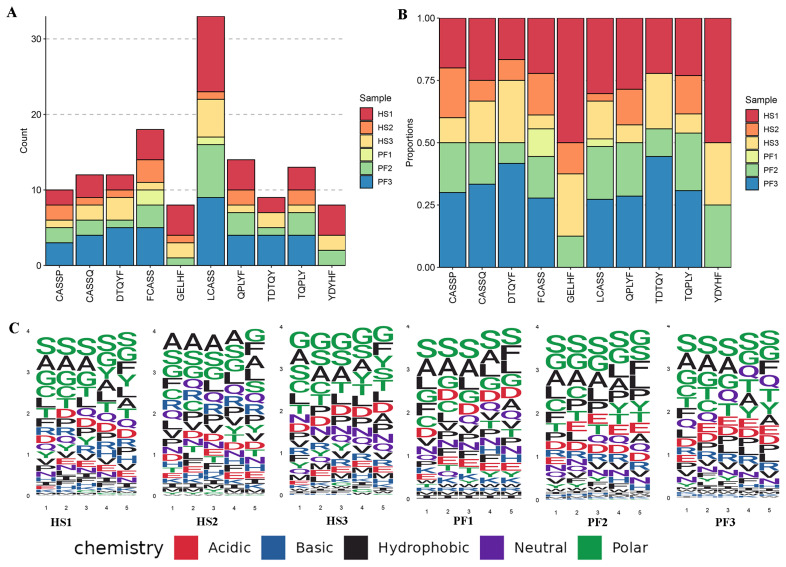
Kmer and sequence motif of cattle liver TCR β CDR3 repertoire. (A) count of top 10 5-mers in sequence length; (B) the distribution of top 10 5-mers in sequence length; (C) position self-information matrix from sequence motif analysis. TCR, T-cell receptor; HS, heat stressed cattle; PF, pair-fed cattle.

Specific Kmers in bovine TCR β CDR3 sequences may be associated with HS status, demonstrating both inter-individual sequence variability and conservation. To identify recurrent patterns or shared segments within the TCR CDR3 repertoire of amino acid sequences, we filtered Kmers of length 5 and conducted statistical analysis on the top 10 Kmers by frequency. Notably, across 6 samples, LCASS exhibited the highest frequency, significantly surpassing FCASS, which ranked second (Figure 4A). Further comparative analysis of the top 10 Kmers between the HS and PF groups revealed the prevalence of GELHF and YDYHF predominantly in the HS group, while other Kmers displayed minimal variation between the two groups (Figure 4B). This suggests a potential association of GELHF and YDYHF with HS in cattle, while the remaining Kmers possibly represent conserved sequence patterns within the T-cell repertoire of liver tissues. Subsequently, leveraging the Kmers’ outcomes, we performed motif analysis based on amino acid composition (Figure 4C), unveiling a higher conservation of motifs in the PF group compared to the HS group. Additionally, polar, and hydrophobic amino acids exhibited relatively higher frequencies within these Kmers; however, their sequence of occurrence and arrangement displayed individual variability.

## DISCUSSION

The liver, a central metabolic organ, exhibits heightened sensitivity to HS [22]. Concurrently, it serves as a pivotal frontline immune tissue, proficient in both immune surveillance by filtering blood and immune clearance of molecules and pathogens originating from the circulation and the gut [5,23]. Evidence supports the liver’s role in HS -induced modulation of both innate and adaptive immune responses, thereby exerting adverse effects on the mammalian immune system [24,25]. Heat stress profoundly affects livestock health, yet comprehensive investigations into immune system within bovine liver tissues under these conditions remain limited. T cells presence in abundance in liver contributes significantly to immune surveillance and regulatory functions within the hepatic milieu, playing a key role in controlling pathogen infections, tumor immunity, and sustaining immune tolerance [26–28]. Moreover, evidence from both *in vitro* and *in vivo* studies supports the liver cells’ capability to directly engage in antigen presentation to naive T cells, thereby initiating T cell activation [29,30]. This study’s focus on the liver-specific T cell immune repertoire in bovine liver tissue under HS addresses a critical gap in understanding cattle’s adaptive immune response to thermal challenges.

Based on the differential TCR chains, T cells are categorized into γδ T cells and αβ T cells. Unlike humans and mice, where αβ T cells constitute over 95% of peripheral T cells, ruminants, including cattle, exhibit a notably abundant population of γδ T cells, potentially accounting for 60% of the circulating lymphocyte pool in young calves [31]. These γδ T cells actively participate in ruminants’ immune responses to viral infections and inflammatory reactions. Consequently, many scholars argue that unlike in humans and mice, ruminants rely primarily on γδ T cells for their immune responses [32,33]. This study identified the presence of both γδ T cells and αβ T cells in bovine liver tissues, with αβ T cells exhibiting the highest detection rate. This observation suggests a potentially significant role of αβ T cells in immune regulation and maintenance of immune homeostasis within bovine liver tissues. Moreover, it indicates a possible disparity in the current understanding of the functional role of T cell subsets in cattle, necessitating further research to comprehensively comprehend the immunological functions and specific mechanisms of these two T cell subsets across ruminants, humans, and mice.

The lengths of the TCR beta chain’s CDR3 region exhibit variation across species [34–36]. Typically, in humans, the peak lengths of the TCR beta chain’s CDR3 region occur at 14 and 15 amino acid residues, while in mice, it occurs at 12 and 13 residues. However, this study observed that in cattle, the peak lengths of the TCR beta chain’s CDR3 region primarily occurred at 15 and 16 amino acid residues. This divergence in CDR3 lengths might reflect species-specific characteristics in immune response and antigen recognition. Moreover, the shortened CDR3 length observed in the HS group compared to the PF group may be associated with its involvement in the immune response, indirectly indicating that HS triggers an immune response in the liver of cattle. However, further research is needed to deeply investigate the implications of this length distribution on the functionality and immune modulation within the cattle immune system, as well as to delineate immunological discrepancies between cattle and other species.

The liver is considered to be an organ with unusual immune functions promoting immune tolerance rather than immunity [37]. The present study has uncovered an intriguing phenomenon: under conditions of HS, the diversity of the T-cell receptor repertoire within bovine liver tissues surpasses that of the PF group. This observed augmentation might be attributed to the liver’s endeavor to maintain internal homeostasis amidst heightened workload, wherein T-cells present in the venous blood traversing the liver are recruited to this organ, thereby contributing to the heightened diversity of the T-cell repertoire within the hepatic milieu.

The ability of T cells to access tissues typically requires multiple steps of T cell extravasation/migration that are regulated by the expression of specific adhesion and homing molecules [38]. However, such a process is not strictly necessary in the liver where the lack of a continuum basal membrane separating the blood from the hepatic parenchyma allows direct T cell scanning of target cells through the fenestrated liver sinusoidal endothelial cells [39]. Based on the expression of CD69, hepatic CD^4+^ T cells are classified into three distinct subsets: CD69^−^, CD69^INT^, and CD69^HI^. CD69^HI^CD4^+^ cells were identified as tissue resident CD4^+^ T cells, while CD69^INT^CD4^+^ T cells could be found in the circulation and lymph nodes [40]. Therefore, the observed increased diversity in the HS group in this study is likely attributed to the potential migration of peripheral blood T cells into the liver under HS conditions. However, the specific T cell subtypes involved, their significance upon entry into the liver, and the pathways engaged warrant further investigation.

Immunoglobulin receptors or TCRs may establish specific structural features early in development, subject to further refinement during T/B cells maturation [41]. The polarity and hydrophobicity of amino acid residues in the TCR’s CDR3 profoundly impact its spatial structure, stability, and antibody binding affinity. The β chain of human CD8 cytotoxic T lymphocytes contained an abundance of polar residues [42]. The CDR3 region of adipose tissue CD8^+^ T cells from high-fat diet mice selected less polarized amino acids [43]. Amino acid usage in the CDR3 region is nonrandom with a predominance of charged or polar residues in the TCRA transcript and a majority of glycines in TCRB [44]. The overall amino acid composition of the CDR 3 of the hybridomas was dominated by small polar residues such as Gly, Asn, Ser, Glu and Ala [45]. Our research unveils a prevalence of polar and hydrophobic amino acids in bovine liver TCR CDR3 repertoire, likely reflecting adaptation to organ-specific immune needs. This highlights the influence of amino acid composition on TCR functionality and suggests organ-specific adaptations within cattle liver tissue.

This study defines liver-specific T cells based on the analysis of transcriptomic data and CDR3 sequences. Transcriptomic data reflects the cellular activity at the transcriptional level, while CDR3 sequences characterize the specificity of TCRs. However, this indirect inference method still cannot eliminate potential interference or errors from other immune cell types. The validation of the existence and function of liver-specific T cells in future studies should be achieved through *in vitro* experiments or animal model studies.

## IMPLICATIONS

Our research not only advances our comprehension of the intricate dynamics between immune responses and hepatic tissues under heat stress in cattle but also holds promising implications for practical applications. The identified diverse clonotypes and heightened diversity in T-cell responses provide potential insights into developing biomarkers for assessing and monitoring heat stress in bovine populations. The observed differential VJ gene pairings and utilization variations in V and J genes, coupled with specific high-frequency amino acid 5-mers, offer a foundation for precision livestock farming tools. These findings contribute to the development of strategies aimed at mitigating the adverse effects of heat stress on herd health and performance in the dairy industry.

## Figures and Tables

**Table 1:** Statistics of bovine liver TCR and BCR chains identified by MiXCR

Samples	HS1^1)^	HS2^1)^	HS3^1)^	PF1^1)^	PF2^1)^	PF3^1)^
Total sequencing reads	27,495,017	22,179,226	19,948,048	21,524,026	22,089,570	23,558,493
Reads in clonotypes	7,948	3,305	3,045	3,012	3,523	1,506
TRA chains	27,463	19,922	22,351	20,136	23,410	24,399
TRB chains	4,183	3,603	3,190	2,465	3,526	3,644
TRD chains	602	426	508	452	537	603
TRG chains	773	486	540	510	594	630
IGH chains	1,788	827	719	761	875	596
IGK chains	357	238	209	154	253	144
IGL chains	7,207	3,254	3,212	2,567	3,433	1,633
Final clonotype count	2,172	1,173	1,447	426	1,150	702

TRA chain, TCR α chain; TRB chain, TCR β chain; TRD chain, TCR δ chain; TRG chain, TCR γ chain; IGH chain, immunoglobulin heavy chain; IGK chain, immunoglobulin κ chain; IGL chain, immunoglobulin λ chain.

1) HS, heat stressed cattle; PF, pair-fed cattle.
